# siRNAs targeting the ERK2 signaling pathway and *AML1/MTG8* fusion gene attenuate the differentiation, proliferation and growth arrest in t(8;21) leukemia

**DOI:** 10.1186/1753-6561-6-S6-P29

**Published:** 2012-10-01

**Authors:** Salem Bashanfer, Faisal Mutee Al-Hassan, Rosline Hassan, Olaf Heidenreich, Mohamed Saifulaman Mohamed Said, Narazah Mohd Yusoff

**Affiliations:** 1 Advanced Medical and Dental Institute (AMDI), Universiti Sains Malaysia, Bandar Putra Bertam, 13200, Kepala Batas, Penang, Malaysia; 2 School of Medical Sciences, Universiti Sains Malaysia, 16150, Kota Bharu, Kelantan, Malaysia; 3 University of Newcastle, Northern Institute for Cancer Research, Framlington Place, Newcastle upon Tyne NE2 4HH, UK; 4 Faculty of Applied Sciences, Universiti Teknologi MARA (UiTM) 40450, Shah Alam, Selangor, Malaysia

## Background

The t(8;21) translocation is one of the most frequent chromosome abnormalities associated with acute myeloid leukemia (AML). This translocation generates the (AML1/MTG8) fusion protein causing blockage of the differentiation process. Moreover, constitutive activation of the mitogen-activated protein kinase (MAPK) pathway as a consequence of this translocation results in an increase in the proliferation rate of leukemic cells.

## Materials and methods

To ensure subsequent experiments are clinically relevant, we carried out microarray on clinical samples from patients who manifested the t(8;21) translocation together with the corresponding cell lines (Kasumi-1 and SKNO-1). We carried out microarray, qRT-PCR, gene knockdown by siRNA, flowcytometry and various cell assays for apoptosis and cellular proliferation testing.

## Results

Analyses revealed a number of overlapping differentially expressed genes in the AML clinical samples and their corresponding cell lines. These genes were uploaded to the KEGG database through the DAVID software (v6.7) to carry out pathway analyses of these leukemic cells. Furthermore, gene expression profiles of both clinical samples and their relevant cell lines showed increased expression of *AML1/MTG8* and *ERK2* (*MAPK1*)*.*

Using 100 nM and 200 nM of ERK2-siRNA and siRNA-AML1/MTG8, respectively, siRNAs were transfected by electroporation to knockdown *ERK2* and the fusion gene *AML1/MTG8* individually and in combination in the t(8;21) cell lines. Gene knockdowns were validated by qRT-PCR, and demonstrated successful mRNA suppression by approximately 80% to 90%. In addition, a slight increase of *ERK2* mRNA expression was observed upon *AML1/MTG8* suppression.

FACS experiments showed reduced expression of CD34 cell surface marker when *AML1/MTG8* was knocked down, which indicated onset of the differentiation process whereas the *ERK2* suppression displayed higher CD34 expression when compared with mock and siRNA controls, indicating an antidifferentiation effect. Cell cycle analyses demonstrated increased growth arrest where G_0_/G_1_ phase increased by 10% to 15% individually and 25% to 30% in a combination, which correlated with the MTS assay results. An apoptosis assay revealed anti-apoptotic effect of *ERK2* and *AML1/MTG8* suppression, which was increased when silenced together in a combination silencing.

A subsequent microarray experiment on cell lines with these silenced genes was consistent with previous results and disclosed several genes that might be involved in controlling these processes.

## Conclusions

These results suggest possible roles of *ERK2* activation in differentiation induction, while *ERK2* inhibition arrested cell growth and reduced proliferating cells in t(8;21) leukemia when combined with *AML1/MTG8* knockdown.

